# A Comparative Evaluation of Inertial Sensors for Gait and Jump Analysis

**DOI:** 10.3390/s21185990

**Published:** 2021-09-07

**Authors:** Isaia Andrenacci, Riccardo Boccaccini, Alice Bolzoni, Giulio Colavolpe, Cosimo Costantino, Michelangelo Federico, Alessandro Ugolini, Armando Vannucci

**Affiliations:** 1 Department of Engineering and Architecture (DEA), University of Parma, 43124 Parma, Italy; isaia.andrenacci@studenti.unipr.it (I.A.); riccardo.boccaccini@studenti.unipr.it (R.B.); alice.bolzoni1@studenti.unipr.it (A.B.); michelangelo.federico@studenti.unipr.it (M.F.); alessandro.ugolini@unipr.it (A.U.); armando.vannucci@unipr.it (A.V.); 2 Department of Medicine and Surgery, University of Parma, 43126 Parma, Italy; cosimo.costantino@unipr.it

**Keywords:** inertial measurement unit, wearable sensors, gait analysis

## Abstract

Gait and jump anomalies are often used as indicators to identify the presence and state of disorders that involve motor symptoms. Physical tests are often performed in specialized laboratories, which offer reliable and accurate results, but require long and costly analyses performed by specialized personnel. The use of inertial sensors for gait and jump evaluation offers an easy-to-use low-cost alternative, potentially applicable by the patients themselves at home. In this paper, we compared three inertial measurement units that are available on the market by means of well-known standardized tests for the evaluation of gait and jump behavior. The aim of the study was to highlight the strengths and weaknesses of each of the tested sensors, considered in different tests, by comparing data collected on two healthy subjects. Data were processed to identify the phases of the movement and the possible inaccuracies of each sensor. The analysis showed that some of the considered inertial units could be reliably used to identify the gait and jump phases and could be employed to detect anomalies, potentially suggesting the presence of disorders.

## 1. Introduction

Gait analysis is fundamental to identify the occurrence of disorders that affect motor behavior, by means of the study and evaluation of different sets of motion parameters. Accurate tests are usually performed in specialized laboratories by exploiting complex and expensive instruments, which require qualified personnel to operate. For these reasons, although reliable and accurate, analyses performed in a laboratory are unavailable for most of the clinical and research communities [1].

The technological advancement in the field of inertial sensors, however, has provided an alternative solution. The benefits of sensor-based motion analysis are many fold: the sensors are low-cost, easy-to-use devices, which can be potentially applied in every environment and by almost anyone, without specific training. In particular, analyses can be easily performed at home, where data can be collected by means of a simple computer, and then subsequently processed and analyzed offline. We refer the reader to the papers [2,3] and the references therein for a review of the most recent results on the use of inertial sensor for motion analysis.

Inertial sensors are usually embedded in inertial measurement units (IMUs), devices including multiple different sensors (gyroscope, accelerometer, magnetometer), whose collected data can be combined to obtain precise motion measurements. In this paper, we compare three of the several IMUs that are available on the market, with the specific purpose of evaluating their performance by means of a set of standardized tests for the evaluation of gait and jump motions. Our aim was to identify and highlight the advantages and disadvantages of each of the considered IMUs in different tests and scenarios, by means of a comparison of data gathered on two healthy subjects.

The paper is organized as follows. Section 2 introduces and describes the three IMUs considered in this study. Section 3 presents a series of typical physical tests, commonly adopted in medicine and rehabilitation. Section 4 details the methods adopted in this work to evaluate the collected data, by providing mathematical and physical models for each test. Section 5 presents the full set of results we obtained in the different tests, together with some comments and comparisons. Finally, Section 6 concludes the paper.

## 2. Tested Wearable Sensors

**Figure 1:**
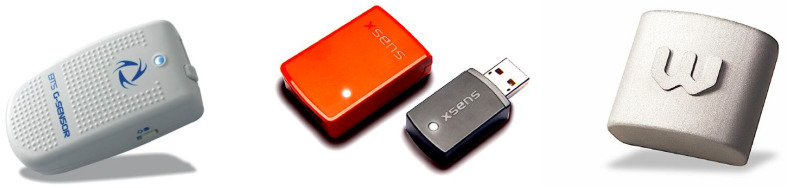
Image of BTS (**left**), MTw (**middle**), and WIVA (**right**).

Among the different sensor platforms that are commercially available, we selected the three wearable sensors that are shown in Figure 1. Their technical specifications are detailed hereafter, along with the main differences that characterize their ease of use and adaptability to the purposes of this study. In this respect, data acquisition protocols play a major role. As it is intuitive, a medical facility would not be interested in accessing the raw data measured by the sensor; rather, a preprocessed and formatted dataset would be useful for diagnostic purposes. A system engineer would instead be interested in a more transparent acquisition of measured data, to be processed offline for the purpose of investigation.

All of the tested sensors were indeed inertial measurement units (IMUs) containing different sensing elements such as accelerometers, gyroscopes, magnetometers and possible other data acquisition devices.

### 2.1. XSENS “MTw Awinda”

The MTw Awinda sensor (from here on referred to as “MTw”) provided by the XSENS company is an inertial-magnetic measurement unit (IMMU), composed of five devices [4]: a normal accelerometer, a gyroscope, and a magnetometer, together with a barometer and a thermometer, in order to provide an autonomous self-calibration procedure every 60 s of use. In contrast to traditional inertial sensing architectures, where a lower output rate results in the degradation of precision, XSENS developed a so-called strap-down integration (SDI) algorithm that guarantees, in dynamic conditions, a high accuracy irrespective of the output data rate. The collected data are first filtered by a low-pass filter (with a cutoff frequency equal to 184 Hz), which reduces the sample frequency, then the calibrated signals are processed by the SDI algorithm so as to calculate and output orientation and velocity increments at a frequency selected by the user. The maximum sampling frequency equals 1 kHz and is the largest among the three tested sensors. The device is equipped with a buffer of memory that can hold up to 1000 samples and, joined with the SDI algorithm, prevents loss of information. In order to reduce the drift error both in dynamic and in static acquisition mode, XSENS implemented a Kalman filter that allows the computation of orientation data directly on board [5]. Moreover, a proprietary radio protocol called Awinda has been designed. This allows detecting intervals in real time and retransmitting missed data packets during the acquisition, so that the accuracy and integrity of data are preserved even in the case where data packets would be lost [6]. The data acquisition frequencies allow more than one sensor to be used at the same time, despite the resulting latency being proportional to the number of used sensors and being able to increase up to 19 ms. The allowed maximum frequency is reported in Table 1 as a function of the number of connected sensors:

The measurement ranges of the accelerometer and gyroscope are:

- Accelerometer range ±160m/s^2^;
- Gyroscope range ±2000degree/s.

Data transfer from the MTw exploits WiFi technology, and the sensors can be connected to a proprietary software or to the widespread scientific computation software MATLAB.

Remarkably, the XSENS MTw is the only IMU that has an API library in C-code, which is called XDA and is used by a large community. The API library can be used to implement MATLAB code to perform data acquisition without accessing proprietary software (which is usually limiting) or live data analysis. In fact, all data from the MTw within this study were processed with a self-made MATLAB code, which embeds the acquisition of all the necessary data: raw accelerometer, gyroscope, magnetometer, and orientation data (both in the form of Euler angles and quaternions). The sensor was applied to the body following the ISB recommendations for standardization [7].

### 2.2. Letsense “WIVA”

The WIVA is a wearable sensor manufactured by Letsense Group, with which it is possible to perform functional evaluations of the movement. Its main technical features are:

- Battery: 3.3 V;
- Autonomy: 12–14 h;
- Communication interface: Bluetooth 4 Low Energy;
- Dimensions: 35×37×15 mm;
- Weight: 50 g;
- Frequency: up to 1000 Hz;
- Output frequency: 100 Hz;
- Usage temperature: 0 ${}^{\circ }$C to 60 ${}^{\circ }$C;
- Accelerometer sensitivity: ±15 m/s^2^;
- Gyroscope sensitivity: 300 degree/s.

This device is equipped with a dedicated software called Biomech to perform gait analysis. Within this study, all data from the WIVA sensor were acquired through its proprietary software, since it is not possible to interface it with a third-party software. During data acquisition, the “Free Test” mode was set in order to obtain both accelerometer and gyroscope data.

### 2.3. BTS “G-Walk”

The G-walk sensor produced by the BTS company [8] (BTS) is strictly focused on the medical field. In fact, it is equipped with a proprietary software that provides several types of movement analysis and diagnostics. It features a triaxial accelerometer with 16 bit quantized data per axis and a manageable acceleration sensitivity. The triaxial gyroscope also uses 16 bit quantized data per axis with adjustable sensitivity. It uses Bluetooth technology for wireless data communication, with a maximum range of 60 m. All data from this sensor were acquired with its proprietary software, called G-Studio, which employs Free4Act technology. As done for the WIVA sensor, we set the “Free Test” mode for data acquisition, in order to obtain accelerometer, gyroscope, and orientation data (which are represented only by Euler angles). Although BTS has a built-in magnetometer, the proprietary software does not allow the acquisition of its data.

The measurement ranges of accelerometer and gyroscope for this sensor are:

- Accelerometer range ±20, ±40, ±80, ±120 m/s^2^;
- Gyroscope range ±250, ±500, ±1000 degree/s.

## 3. Tests in Physical Medicine and Rehabilitation

In order to compare the inertial sensors from a medical point of view, four different tests were carried out. Two of them, called “10 m Walk” and “Timed Up and Go” (TUG), are related to the human walking pattern, while the other two, called “Counter-Movement Jump” (CMJ) and “Stiffness” test, are related to the human jumping pattern. In addition, a fifth “Control Test” was inserted before the two jumping tests, with the aim of achieving a reliable model for further data analysis, as discussed below.

### 3.1. The 10 m Walk Test

This test allows a rapid and precise analysis of the walking sequence and represents an ideal diagnostic tool for the evaluation of many neurological and orthopedic pathologies that can adversely affect the walking pattern of a patient. It is thus largely used in the medical field to support the diagnosis with various important parameters of human walking such as the duration of the gait cycle phases, the average speed of a patient, the cadence of steps, and the step length. Other meaningful parameters are: the walking symmetry index, which quantifies the subject’s ability to accelerate the center of mass in either way during the cycle of right–left steps; the walking quality index, which measures the ability of the subject to split his/her gait cycle correctly between the support phase and the swing phase; the propulsion index, which describes the patient’s ability to fully accept body weight on a limb after the deceleration phase and push the center of mass forward onto the opposite limb.

**Figure 2:**
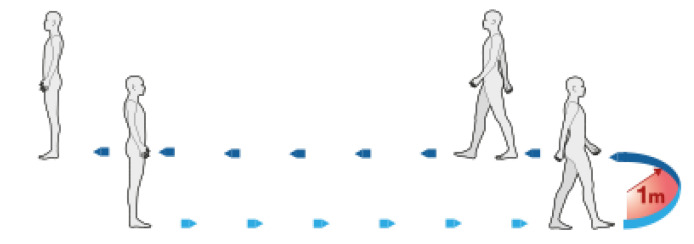
10 m Walk Test.

The test consisted of the subject walking 10 m in a straight line, then a change of direction took place with the subject walking around a cone in order to return along a straight line to the starting point. The subject must start from an immobile upright position (orthostatic position), as shown in Figure 2.

**Figure 3:**
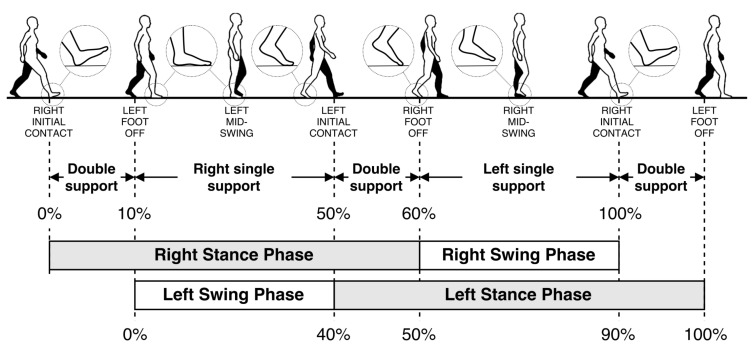
Graphical representation of the gait phases (image from [9]).

Generally, human walking is a periodic movement of body parts with repetitive motions. To better understand this periodic walking sequence, the gait phases can be used to describe a complete walking pattern and are graphically represented in Figure 3.

Detailed definitions of the gait phases are as follows:

- Initial contact (IC): This phase, also called heel strike (HS) in standard notation, comprises the moment when the foot touches the floor. The joint postures presented at this time determine the limb’s loading response pattern;
- Loading response: This phase, also called foot flat (FF) in standard notation, is the initial double-stance (DS) period. The phase begins with initial floor contact and continues until the other foot is lifted for swing;
- Midstance (MM): This phase is the first half of the single-limb support interval. Here, the limb advances over the stationary foot through ankle dorsiflexion, while the knee and hip extend. Midstance begins when the other foot is lifted and continues until body weight is aligned over the forefoot;
- Terminal stance: This phase, also called heal off (HO) in standard notation, completes the single-limb support (SS). The stance begins with the heel rising and continues until the other foot strikes the ground. Throughout this phase, body weight moves ahead of the forefoot;
- Pre-swing: this final phase, also called toe off (TO) in standard notation, is the second double-stance interval in the gait cycle. Pre-swing begins with the initial contact of the opposite limb and ends with the ipsilateral toe off. The objective of this phase is to position the limb for swing;
- Initial swing: This phase is approximately one-third of the swing period, beginning with a lift of the foot from the floor and ending when the swinging foot is opposite the stance foot. In this phase, the foot is lifted, and the limb is advanced by hip flexion and increased knee flexion;
- Midswing: this phase begins as the swinging limb is opposite the stance limb and ends when the swinging limb is forward and the tibia is vertical;
- Terminal swing: This final phase of swing begins with a vertical tibia and ends when the foot strikes the floor.

Each gait phase has a functional objective and a critical pattern of motion. The sequential combination of the phases enables the limb to accomplish three basic tasks: weight acceptance, single limb support, and limb advancement.

### 3.2. The Timed Up and Go Test

**Figure 4:**
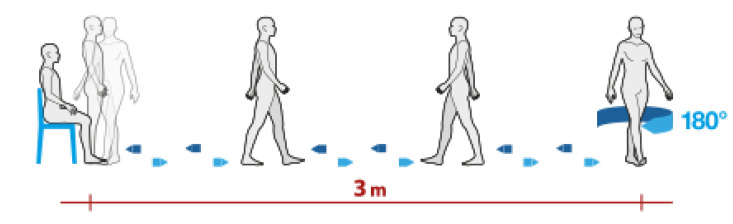
Graphical representation of the TUG test.

The Timed Up and Go (TUG) is a simple, quick, and widely used clinically approved test for the measurement of mobility and fall risk that was first introduced in 1991 by Podsiadlo and Richardson [10]. The authors described the instructions to be given to the subject of the test as follows: “rise from a standard arm chair, walk to a line on the floor 3 m away, turn, return, and sit down again.”, as graphically depicted in Figure 4. As in the 10 m Walk Test, a cone is usually placed at the point where the subject has to turn around.

The TUG Test is widely used in the examination of the elderly, but is also becoming important in other analyses [11]. It is divided into the following phases:

- Sit-to-stand time (SITS): the time taken by the subject to stand up from a sitting position. This phase is further divided in two parts: in the first part (flexion phase), the subject prepares himself or herself to stand up by carrying out a forward flexion of the torso; in the second part (extension phase), the subject stands up by extending the torso, which returns to the vertical position;
- Outward and return journey times: the time taken by the subject to walk from the chair to the cone in a straight line (outward) and vice versa (return);
- Turn-around time (TA): There are two different TA times: the first is the time it takes the subject to walk around the cone; the second is the time it takes the subject to turn around before sitting down again;
- Stand-to-sit time (SSTS): the time taken by the subject to sit down from a standing position. This phase is also divided in two parts: in the first part (flexion phase), the subject prepares himself or herself to sit down by carrying out a forward flexion of the torso; in the second part (extension phase), the subject sits down by extending the torso and returning to the vertical position.

The TUG Test has already been performed with inertial sensors in other studies, by Gina Sprint et al. [12], Aner Weissa et al. [13], and James Beyea et al. [14].

### 3.3. A Control Test

The Control Test, which we introduced for calibration purposes, simply consists of jumping to the ground from a platform of known height. Such an extra test turned out to be necessary in order to check the accuracy of the data processing that was later applied to the “Counter-Movement Jump” and to the “Stiffness” tests.

In particular, for the Control Test to be effective, a correct processing of the data must be such that the estimate of the vertical distance from the platform to the final landing position must coincide with the platform. We describe the appropriate signal processing for this test in Section 4.3.

### 3.4. The Counter-Movement Jump Test

**Figure 5:**
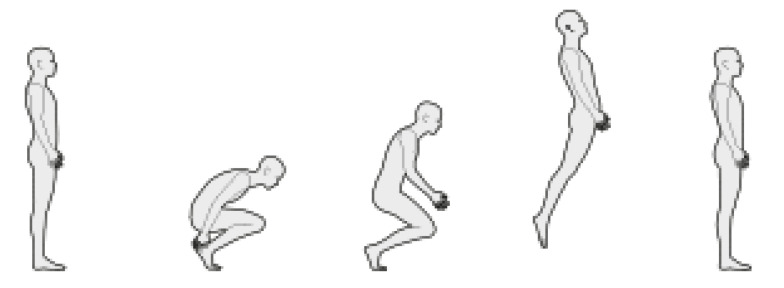
CMJ Test.

The jump with counter-movement (CMJ) starts by maintaining an upright position, with the hands resting on the hips and the feet close to the floor and arranged at a width equal to that of the shoulders. A quick 90${}^{\circ }$ bend is performed with the legs, and then, a jump in the vertical direction is taken, as in Figure 5. During the flight phase, it is not allowed to flex the knees; the hands should not loose their position; the relapse must be performed with extended knees, with subsequent cushioning to avoid trauma. To prevent the test from being compromised, it is important that during the push-up action, the torso remains as straight as possible. At the moment of landing, the two feet must touch the ground at the same time. This is a functional test for a dynamic analysis, able to provide quantitative and objective information for the various phases of the jump.

### 3.5. The Muscle Stiffness Test

The last test that was considered concerns muscle stiffness. During the test, the subject performs 10 consecutive jumps. Starting from a standing position, keeping the feet aligned with the shoulders, heels close to the ground, and arms at the sides for the whole duration of the test. The patient prepares for the jump by flexing his or her legs at the start of every jump, but keeping them taut during flight and upon landing. In this test as well, it is important to try to keep the torso as straight as possible.

## 4. Data Analysis and Methods

Each of the medical tests described in the previous section involves different movements and requires the sensors to be attached to specific critical points, in order to characterize the movements. In addition, only some of the measured data (e.g., linear acceleration, Euler angles, gyroscope data, etc.) are relevant to isolate and measure specific phases of given movements. Finally, proper processing of each data stream is always advisable and sometimes strictly necessary in order to remove artifacts or to enhance the visibility of distinctive features.

Although little investigated in the literature, the position of the sensor has been recognized to influence the acquired data and the harmonic content of the recorded signal, sometimes requiring specific postprocessing [15,16]. We did not investigate this issue, which was outside the scope of our study, and in our experiments, we always followed the prescriptions of the sensors’ manufacturers.

For each of the performed tests, we describe in the following the data acquisition and processing methods that were implemented.

### 4.1. 10 m Walk

A first necessary operation on the acquired data, which is needed to bring the raw acceleration data from the sensor’s frame of reference to that of the Earth, is the elimination of the gravitational component that affects the *Z* axis. The vertical acceleration data were of even greater importance for the experiments that follow (see Section 4.4 and Section 4.5), which involved jumps. Thus, we analyze this problem in depth in Section 4.3, where different solutions are proposed for the different sensors. As explained therein, the elimination of the gravitational acceleration was feasible for the MTw and BTS sensors, which provides the necessary orientation data, but not for the WIVA sensor, which does not.

**Figure 6:**
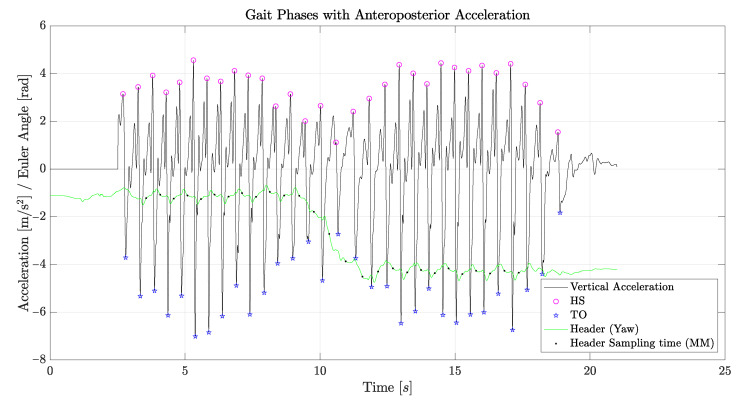
Gait phases obtained from the anteroposterior accelerometer of the BTS sensor.

Step detection through the acceleration data from the lower trunk can be carried out in two different ways, by observing either the anteroposterior acceleration or the vertical acceleration. If the anteroposterior acceleration is taken into account, as reported in Figure 6, each peak corresponds to a heel strike (HS), and the following minimum corresponds to the toe off (TO) of the other foot. With these two phases, the midstance (MM) of every step can also be established.

**Figure 7:**
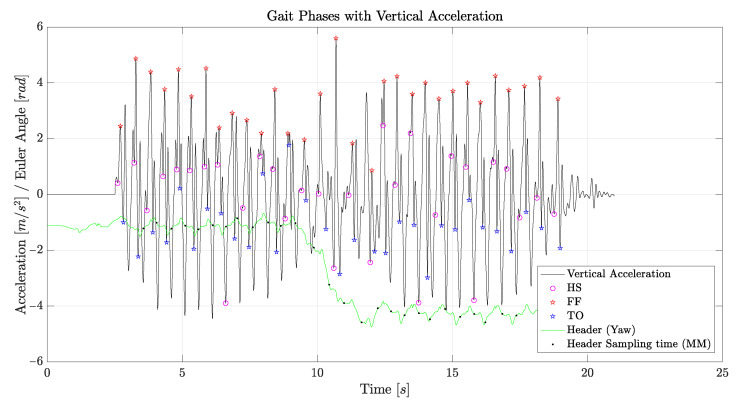
Gait phases obtained from the vertical accelerometer of the BTS sensor.

If instead the vertical acceleration is taken into account, as reported in Figure 7, each peak corresponds to a foot flat (FF); the previous minimum corresponds to the heel strike (HS) of the same foot; the following minimum corresponds to the toe off (TO) of the other foot. In this case, midstance (MM) can also be evaluated, and knowing the FF times, double support phases can be established as well. It is therefore clear that vertical acceleration data should be preferred. From mediolateral acceleration, it is possible to discriminate between left and right steps: the lower trunk accelerates shifts to the left during a right support phase and to the right during a left support phase. However, in the present analysis, discrimination between right and left steps was established using the gyroscope data, which appeared to be much more reliable. Figure 6 and Figure 7 also report the header, representing the rotation of the subject around his or her vertical axis.

There are two main methods to evaluate the step length (SL). The first is based on the maximum and minimum vertical acceleration $\left(a_{z,\max},a_{z,\min}\right)$ of the lower trunk [17]:

(1) $$\mathrm{SL}=K\sqrt[{4}]{a_{z,\max}-a_{z,\min}}\;,$$

where *K* is a subject-dependent calibration constant. The second method is based on the “inverted pendulum model” (IPM) of gait [18,19,20]. The IPM exploits four different equations for the evaluation of the SL, which have already been compared in various studies [18]. In this work, we adopted the one that does not need calibration factors:

(2) $$\mathrm{SL}=2\sqrt{2lh-h^{2}}+S\;,$$

where *l* is the length of the leg, *h* is the maximum displacement of the lower trunk during a step cycle, and *S* is the foot length.

In order to calculate the vertical displacement, a double integration over time of the acceleration data is needed. An integration over the entire walking time is however out of question, since the drift error would make the result totally unreliable. Luckily, the vertical velocity obeys known conditions: it is null at the FF time instants ${\mathrm{FF}}_{i}$; hence, the integration interval can be split into separate steps (from ${\mathrm{FF}}_{i}$ to ${\mathrm{FF}}_{i+1}$),

(3) $${\tilde{v}}_{z}\left(t\right)=\int_{t_{{\mathrm{FF}}_{i}}}^{t}a_{z}\left(\tau \right)d\tau \;\left(t_{{\mathrm{FF}}_{i}}<t\leq t_{{\mathrm{FF}}_{i+1}}\right)$$

and velocity can be corrected with a linear estimation of the drift:

(4) $$v_{z}\left(t\right)={\tilde{v}}_{z}\left(t\right)-\left(a_{0}+a_{1}t\right)\;.$$

where $a_{0}$ and $a_{1}$ can be calculated from the application of initial and final conditions (${\tilde{v}}_{z}\left(t_{FF_{i}}\right)=0$ and ${\tilde{v}}_{z}\left(t_{FF_{i+1}}\right)=0$), as further discussed in Section 4.3. The corrected velocity $v_{z}\left(t\right)$ can thus be integrated over time to yield the vertical displacement *h* that is needed in Equation (2).

Data recordings were carried out on two different healthy subjects: a 34-year-old male and a 24-year-old female. Subjects were asked to walk at their natural speed. The sensors were positioned below the line that connects the two dimples of the Venus-lumbosacral passage, which corresponds to the S1-S2 vertebrae, and were firmly attached to the body with the supplied straps. Every measurement was taken six times with each of the three sensors for the male patient, while only the BTS and MTw sensors were measured for the female patient. The reason was that the data from the WIVA sensor turned out to be considerably less accurate, compared to the other two, as further discussed in Section 5.1. The duration of outward and return journey and the duration of turn were measured with a stopwatch.

### 4.2. Timed Up and Go

The analysis of this test only requires the data from the Euler angles or from the gyroscope. While the analysis can be easily accomplished when using orientation data, it becomes more difficult when resorting to the gyroscope data, whose reliability is typically poor.

The duration of the entire test was measured with a stopwatch. To obtain the initial and final moments of SITS and SSTS phases, the pitch angle (the Euler angle corresponding to a rotation of the patient around his or her mediolateral axis) must be analyzed. Indeed, it can be noticed that the pitch angle varied significantly only in the rising and sitting phases, in which the sensor attached to the body followed the rotating motion described in the previous section. Similarly, turning phases can easily be found by observing the yaw angle (heading of the patient). Orientation data were filtered to obtain cleaner graphs to work on, in a tradeoff between the suppression of vibrational noise and the integrity of the information related to movement. A good compromise was found by low-pass filtering the pitch angle data with a cut-off frequency of 2.3Hz and the yaw angle data with a cut-off frequency of 0.85Hz.

**Figure 8:**
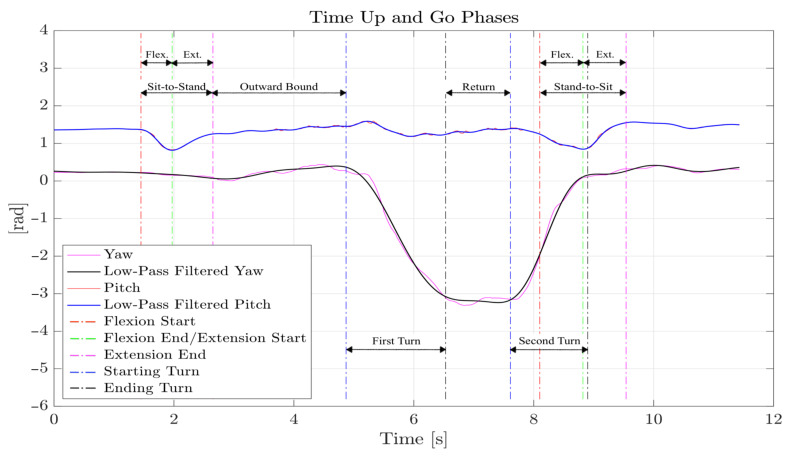
TUG phases obtained from MTw data (male subject).

Figure 8 shows an example of the data acquisition for the TUG phases described in Section 3.2. The data in Figure 8 were captured by the MTw unit. With such reliable data, the critical points to discriminate the different phases can be easily found, both visually and in an automated postprocessing (e.g., with the aid of a MATLAB code). As seen in the Figure, the second TA and the SSTS phase usually overlap, which is a known feature of the TUG Test phases.

As for the 10 m Walk Test, data recordings were carried out on two different healthy patients: a 34-year-old male and a 24-year-old female. Each of the subjects was asked to carry out the test at different speeds. The sensors were positioned above the iliac wings, which corresponds to the L2 vertebra, and were firmly attached to the body with the supplied straps. Every measurement was taken five times with each sensor and for each patient, although the WIVA sensor data resulted in being too difficult to manage (again due to the lack of orientation data).

### 4.3. Control Test

The first problem encountered in analyzing the data provided by the Control Test was the need to switch from the local sensor reference to the global terrestrial reference system, in order to eliminate the gravitational component of acceleration and to effectively calculate the maximum vertical height of the jump. In the absence of this correction, the position relative to the *Z* axis of the sensor would be calculated rather than that relative to the terrestrial one.

Orientation data are needed to calculate the rotation matrix that is used for the transition to the global system. Once in this reference system, the gravitational component can be eliminated simply by subtracting its value (9.80427 m/s${}^{2}$, in Parma, Italy, i.e., the site of the experiments) from the vertical acceleration. As already recalled, orientation data are available both for the MTw (in the form of the Euler angles or quaternions) and for the BTS (only available as the Euler angles), but not for the WIVA sensor. For this specific sensor, we managed to eliminate the constant acceleration components (among which is the gravitational one) with the application of a high-pass filter, namely a digital IIR filter of the Butterworth type, with a 0.1Hz cutoff frequency. Despite it being impossible to switch to a global reference system for the WIVA sensor, we found that the adopted method yielded an acceptable solution to effectively use the vertical acceleration component of the sensor. In any case, acceleration data were filtered with a 4th-order low-pass Butterworth filter with a cutoff frequency equal to 12Hz, in order to obtain a smoother profile and eliminate vibrational noise.

**Figure 9:**
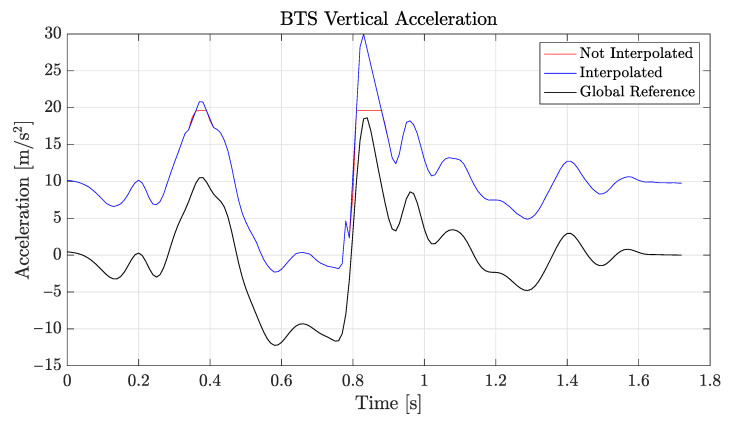
Interpolation of the vertical acceleration (BTS sensor limited to 20 m/s${}^{2}$ maximum acceleration).

Another significant problem encountered during the Control Test was that the WIVA is capable of recording a maximum acceleration value equal to 20 m/s${}^{2}$. The BTS, instead, can be set to record different maximum acceleration values, as reported in Section 2, and the MTw supports the largest acceleration values among the three IMUs. For the WIVA, such a clipping of measured data results in a very large cumulated error in the estimation of the vertical position, which is obtained by (doubly) integrating the accelerometric data. As a partial solution to this technical limit, we linearly interpolated the accelerometric data of the WIVA sensor in the following way. We replaced the clipped part of the signal linearly increasing/decreasing data: the slope of the first (e.g., increasing) part was obtained by the first cut sample and that located two sampling epochs ahead of it; the slope of the second (e.g., decreasing) part was similarly obtained from the last cut sample and that located two sampling epochs after it. The result is graphically reported as an example in Figure 9, for the BTS, where we set the maximum recorded acceleration to 20 m/s${}^{2}$, in order to have a fair comparison with the WIVA. Of course, the resulting data sequence showed a cusp at the crossing of the two interpolation lines, which was however not a problem, although not being physically realistic, since it corresponded to an abrupt inversion of the acceleration derivative. By testing different choices of samples on which to build the linearly interpolated data chunks upon, we found that the samples mentioned above had the best tradeoff in terms of accuracy and versatility. With the introduced interpolation technique, we noticed that the results were much more reliable, although with possible residual inaccuracies that were obviously due to a part of the signal being unknown and artificially reconstructed, as seen in Figure 9.

In order to calculate the vertical speed (and, from that, the vertical position), a numerical integration of the vertical component of acceleration is required. This produces an estimate of sacral vertical velocity (v), which is known to be affected by a drift error. This is a well-established phenomenon in inertial sensors, which constitutes a third (and final) impairment to compensate. Fortunately, the duration of the Control Test was very limited; hence, it is possible to compensate for the accumulation of the drift error by applying a small correction that depends on the known initial and final conditions. Following [21], we applied the following linear approximation for the compensation of the drift error:

(5) $$v\left(t\right)=\tilde{v}\left(t\right)-\left(a_{0}+a_{1}t\right)\;,$$

where $\tilde{v}$ and *v* are the uncorrected and corrected sacral vertical velocities, while $a_{0}$ and $a_{1}$ are constants obtained from the known initial and final sacral velocity of the subject. Finally, to determine the vertical position, *v* was further integrated without applying any correction.

**Figure 10:**
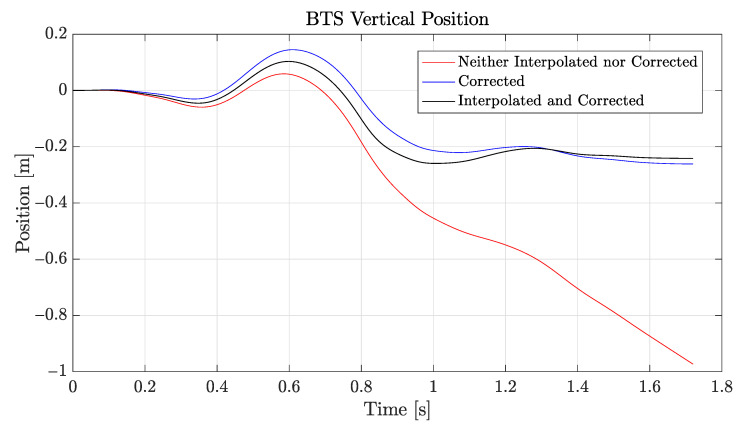
Vertical position (BTS sensor).

Figure 10 shows the vertical position calculated using the raw vertical acceleration (red), the interpolated vertical acceleration (black), or the interpolated and corrected (i.e., with drift error compensation via (5)) vertical acceleration (blue).

For the Control Test, data recordings were carried out on two different healthy patients: a 34-year-old male and a 24-year-old female; each subject was asked to make a small jump from the platform to the ground (17 cm for the male and 16 cm for the female). The sensors were positioned on the last lumbar vertebra, which corresponds to the L5 vertebra, and were firmly attached to the body with the supplied straps. Every measurement was taken two times with each of the sensors described in Section 2, for the first patient, and with the BTS and MTw sensors only for the female patient. We report, in Table 2, the results obtained for the platform position (i.e., the jump height), with or without the interpolation/correction techniques described above:

### 4.4. Counter-Movement Jump

Two of the most important parameters in this test were the maximum height of the jump and the time of flight (TOF). For the calculation of the maximum height, velocity, and vertical position were obtained by applying the same methods discussed in Section 4.1 and Section 4.3. Specifically, the data recorded by the sensors during the test were first imported and analyzed through the MATLAB software. Acceleration data were brought to a global reference through the methods discussed in Section 4.3. After that, data were filtered with a low-pass filter with a cutoff frequency equal to 15 Hz, so as to reduce the vibrational noise and make data processing easier and more efficient.

**Figure 11:**
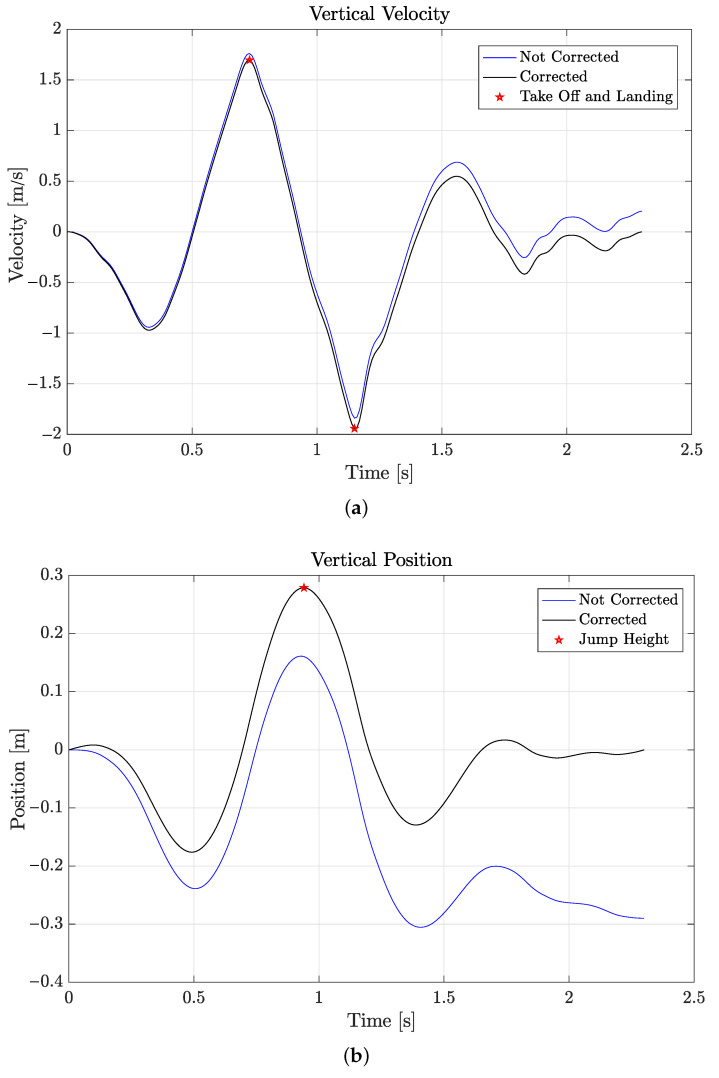
Graphs of: (**a**) vertical velocity and (**b**) position (BTS sensor).

The TOF was obtained by searching the maximum of the vertical velocity and the subsequent minimum, as shown in Figure 11a. As is known from the literature, the TOF equals the time path between these two instants [21,22], as reported in Figure 11b.

For this test as well, data recordings were carried out on two different healthy patients: a 34-year-old male and a 24-year-old female. Sensors were positioned on the last lumbar vertebra, which corresponds to the L5 vertebra, and were firmly attached to the body with the supplied straps. Every measurement was taken three times with each of the sensors described in Section 2 for the male patient and only with the BTS and MTw sensors for the female patient.

As an extra source of information for the CMJ Test, a video was shot during each test, which is a useful resource, for instance, for a deeper investigation of the time of flight. In fact, the TOF value obtained from the data analysis was later compared with that obtained by counting the frames of the video (we used the iMovie program).

### 4.5. Stiffness

For the study of muscle stiffness, it is necessary to focus on the vertical force, which is obtained simply by the second principle of dynamics, F=Ma, where *M* is the patient’s mass and *a* is the vertical acceleration, as recorded by the sensor. Referring to a simple dynamical system with a mass attached to a vertical spring, when the mass is dropped, the elastic force that is transferred from the spring to the mass during landing and take-off is equal to its weight, i.e., to Mg, where *g* is the gravitational acceleration. The time interval between two consecutive points of equilibrium, T/2, equals half of the period of the resonant dynamical system, which is characterized by an angular frequency equal to w=2πT For a patient with mass *M*, which follows such a spring pattern during the repetition of jumps involved in the present test, the stiffness of the muscle–tendon system of the lower limb is calculated as [23]:

(6) $$k=Mw^{2}\;,$$

where *k* is easily seen to be proportional to the patient’s mass and to the square of the resonant angular frequency *w*.

**Figure 12:**
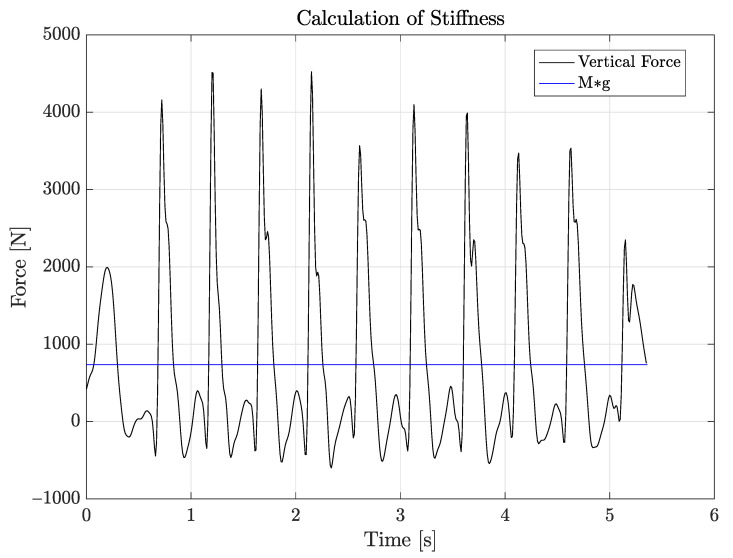
Stiffness calculation graph.

Figure 12 shows the vertical force, as obtained by the measured vertical acceleration, compared to the patient’s weight (horizontal line). The crossing points correspond to the time instants where the vertical force becomes larger or smaller than the weight of the patient [24], and from the abscissas of these points, we can easily measure the period *T*, then the corresponding angular frequency *w*, hence the stiffness parameter *k* in (6).

As for the previous tests, all acceleration data were interpolated, rotated to a global reference system, and filtered through a Butterworth low-pass filter with a 15 Hz cutoff frequency. For this final test as well, data recordings were carried out on the same healthy patients used for the previous tests: a 34-year-old male and a 24-year-old female. As for the CMJ Test, the sensors were positioned on the last lumbar vertebrae and firmly attached with straps. Every measurement was taken three times only with BTS and MTw for both patients; the WIVA sensor was excluded from this test because of the drawbacks shown in the “Control Test”, since it does not provide information on the orientation, which is needed to calibrate and compensate for accelerometric data inaccuracies.

## 5. Results

We shall now illustrate the results obtained for the four test introduced in Section 3, along with some general remarks that apply to all of these tests or to similar ones, in the context of diagnostics in physical medicine.

The tests that were carried out in this work were performed on young healthy patients. Therefore, the collected data do not have any specific medical value. They were rather analyzed for the purpose of comparing the sensors that were the object of this study and to understand if they can provide reliable measurements for the parameters (e.g., time phases) of the tests described in Section 3.

Most of the tests were performed on two subjects only, with a limited number of repetitions and measurements. Hence, although the presented results are not claimed to have any statistical validity, the agreement and consistency of the obtained results for repeated trials on the two different subjects is a sufficient clue to indicate the effectiveness of the proposed measurement protocol.

### 5.1. 10 m Walk

The leg length was measured for the two patients, as the distance from the top trochanter major femoris to the floor. For the male and female patients, a length of 0.92 m and 0.90 m resulted, respectively.

The duration of outward and return journey, as measured by the stopwatch, was compared to the time measurements obtained from the sensors, and no significant difference was found, for any sensor.

As discussed in Section 4.1, two methods can be used to measure the gait phases, resorting to the vertical or to the anteroposterior accelerations, as reported in Figure 6 and Figure 7. Both methods have strengths and weaknesses, in terms of the complexity of the needed data processing. However, the accuracies that can be obtained were comparable, and we did not find significant discrepancies between the numerical results obtained from the two methods.

**Figure 13:**
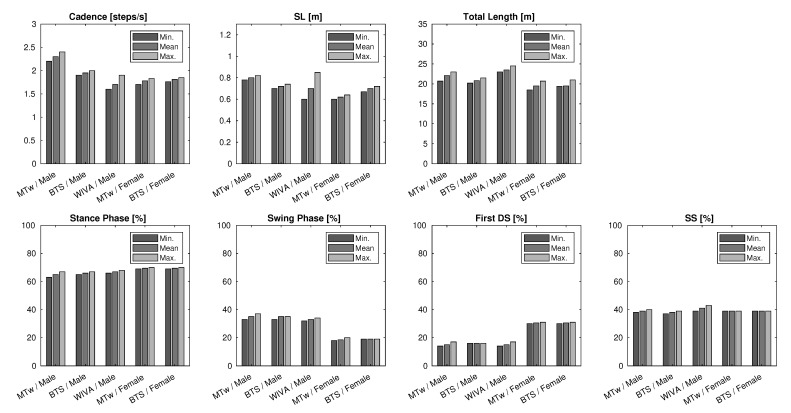
Minimum, mean, and maximum values for the parameters obtained by MTw, BTS, and WIVA in the 10 m Walk Test (6 measurements each).

Figure 13 reports measurement results for the gait phases, obtained from the vertical acceleration of the lower back. The minimum, maximum, and average values, reported in the figure for some of the measured quantities, showed a good repeatability and accuracy of the measurements. Such an accuracy is further witnessed by the data shown in Figure 7, in the case of the BTS sensor, since the data plot can be used to discriminate between all the gait phases very accurately. A similar precision was obtained in almost every measurement performed with each sensor, as confirmed by the results in Figure 13. Indeed, each sensor returned reliable and repeatable data for the gait phases, with slightly less accurate measurements for the WIVA sensor, although still acceptable.

The acquisition and processing of accelerometric data became more problematic, for the WIVA sensor, in the case of data integration, described in Section 4.1. Without the implementation of drift correction, as expressed in (4), the resulting vertical velocity was less accurate and hardly repeatable, for the WIVA sensor; an effect that was not noticeable when using the BTS or MTw sensors. The results in Figure 13, and especially the values of step length (SL) and total path length confirmed this observation. In particular, the quality of measured data was not adequate in the case of the female patient, and unfortunately, it was not possible to repeat the measurements, as discussed in Section 5.6. For this reason, we decided not to process the measurements with the WIVA sensor for the female patient.

From the measurements, we can conclude that the MTw sensor showed the best performance in terms of the accuracy and repeatability of the measured data. The BTS sensor proved its ability to yield accurate data most of the time. In these kinds of measurements, the WIVA sensor succeeded in some tasks, but made the acquisition and processing of data more difficult, due to the strictly needed drift correction.

### 5.2. Timed up and Go

The same patients as in the 10 m Walk Test were asked to perform the TUG Test at three different speeds: fast, medium, and slow. Measurements were repeated five times, for each patient, speed, and each sensor used. Due to the lack of orientation data, the WIVA sensor was excluded from this test.

**Figure 14:**
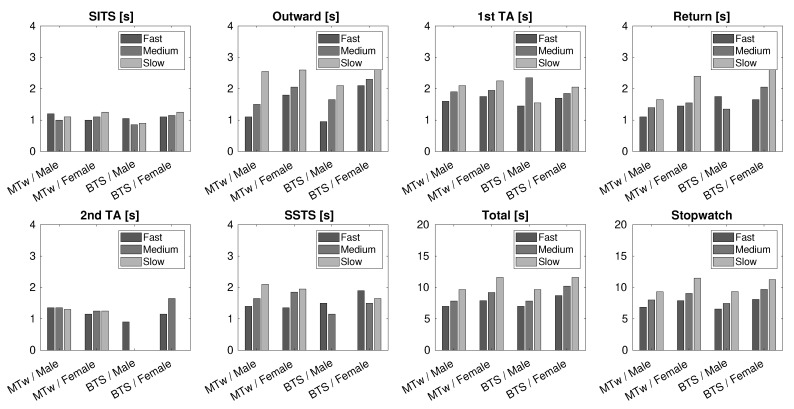
Phase times obtained by the TUG Test, for the two tested sensors (MTw and BTS). All data are in seconds.

Figure 14 reports the measured values of the various time phases of the TUG Test, described in Section 3.2 (all values are in seconds). As was expected, for almost all time phases, the measured values increased from the fast to the slow mode of repetition of the test. Data recorded from the stopwatch (last graph) were very similar to that obtained from the sensors. For the BTS sensor, we marked with a null value (i.e., the bar is absent) those phase times that were absent or whose measurements showed an unrealistic value. These markers highlight those situations where the same data processing algorithms, described in Section 4, that worked for the MTw data did not manage to identify all the TUG phases correctly, when applied to data provided by the BTS sensor. Therefore, for the TUG Test, although BTS often gave convincing results, the reliability of the data provided by the MTw sensor proved to be superior.

### 5.3. Counter-Movement Jump

As described in Section 4.4, for this test, the time of flight was measured first by processing the vertical acceleration data provided by the sensors and then compared by that obtained by video frame counting. The jump height was finally obtained by integrating the (corrected) vertical velocity. Recall that for both the BTS and the WIVA sensors, acceleration data were reconstructed from an interpolation procedure. Before the test, patients were told to jump always at their maximum elevation.

Table 3 shows the data obtained by three repetitions of the test, for each patient and sensor. From Table 3, it can be seen how the average height obtained by the acceleration data from the WIVA sensor was considerably higher that that of the MTw and BTS sensors. This was again due to the difficulties encountered in processing the acceleration data from the WIVA sensor, and for this reason, we disregarded the data for the second patient, which were of poor quality, so that the data were considered with the WIVA sensor for the male patient only. Unfortunately, it was not possible to repeat the measurements for the female patient, as discussed in Section 5.6. For the BTS sensor, the height resulting from the second jump of the male patient was much smaller than the other two. In general, the results obtained from the MTw data were stabler, across the three jumps. This fact should be related to the features of the MTw sensor, whose larger dynamics does not require the numerical interpolation discussed in Section 4.3.

### 5.4. Stiffness

Through the methods discussed in Section 4.5, the value of the resonance angular frequency *w* and that of the resulting stiffness parameter *k* were evaluated for the two usual patients by letting them repeat the test three times. As already discussed in Section 4.5, due to the lack of orientation data on the WIVA sensor, data were collected only with the MTw and BTS sensors.

Table 4 shows the minimum, mean, and maximum values for the *k* and *w* parameters, as measured during the three repetitions of the test, for each patient and sensor used. As for the female patient, the results produced by the MTw sensor were stable for both parameters, with a relatively higher stability for the MTw sensor. A similar situation occurred for the male patient, where the MTw sensor yielded stabler data.

Note that the calculation of the stiffness parameter was based on data that were not sensitive to the interpolation procedure described in Section 4.3, which means that the lower dynamic range (20 m/s${}^{2}$) that we selected for the BTS sensor was not responsible for its relatively lower performance in terms of measurement stability.

### 5.5. General Discussion

In terms of the comparison among the three sensors that were employed—albeit not all of them in every test—the superior performance of the MTw sensor in terms of resilience to drift error appeared quite evident. Especially in the 10 m Walk Test, which lasts for a longer time, sensors tend to accumulate errors, due to the fact that, in general, measurements are correlated in time (typically, each next measurement depends on the actual one and on the previous one). In order to avoid this effect, as mentioned, the MTw sensor implements a Kalman filtering, which allows the reduction of the drift error. Such a solution is not present for the other IMUs, hence their higher susceptibility to drift.

Another aspect that favors the MTw sensor is that it can be used with an external software, such as MATLAB (the XSENS company provides examples on how to use the sensor with different software). The other two sensors, instead, can only be used with their own proprietary software, making data acquisition and processing more difficult. On the other hand, the proprietary software is especially suited for using the sensor in the medical field; hence, the nontechnical user can benefit from pre-engineered functionalities, without the need to implement them personally.

### 5.6. Limitations

This work stems from a cooperation between physicians and engineers. The tested IMUs, described in Section 2, were the only device models available at the time of performing the medical tests and related measurements.

While the medical research group took care of executing the tests, the engineering group took care of the (often offline) processing of the acquired data. For this reason, when the processed data showed an unsatisfactory quality of measured data, it was not always possible to repeat the measurement and raw data acquisition, as in the case of the female patient with the WIVA sensor, whose data are missing in Section 5.1 and Section 5.3.

Since this is an exploratory study on different IMUs for several medical tests, the volume of acquired data was limited, and we concentrated more on developing effective processing techniques for these tests. Hence, the statistical significance of our collected data was limited. A more comprehensive measurement campaign, extending the data acquisition to a much larger sample, is thus left as a future work.

## 6. Conclusions

We discussed the use of several inertial sensors in the field of human motion. The goal was to compare three different platforms in order to establish their suitability in the medical field. We implemented four medical test that were performed with three sensor models, XSENS MTw, Letsense WIVA, and BTS G-walk. The results of the medical tests pointed out that the WIVA sensor is severely impaired by the absence of orientation data. A well-known problem of inertial sensors technology is the presence of drift errors. The correction procedure provided by the software supporting the MTw sensor can drastically reduce drift errors and is of great benefit for the precision of measurements. Moreover, the support given by the XSENS company to the use of third-party software makes this sensor easier to test and manage.

Hopefully, the advancements of inertial sensors technology will overcome most of the drawbacks of today’s devices and make their use simpler and wider. The future of this technology is prosperous and will enable physicians to have diagnostic tools that are fast and reliable to support them in their profession.

## Figures and Tables

**Table 1:** Maximum frequency as a function of the number of sensors for the MTw.

Number of Sensors	Maximum Frequency
1 up to 5	120 Hz
6 up to 9	100 Hz
10	80 Hz
11 up to 20	60 Hz

**Table 2:** Platform height. Male results to the left; female results to the right.

Platform Height (m)	WIVA	BTS	MTw	Platform Height (m)	BTS	MTw
No interpolation	4.2	6.8	0.38	No interpolation	9.3	0.25
Interpolation	0.25	0.22	0.195	Interpolation	0.16	0.205
Corrected	0.145	0.213	0.195	Corrected	0.168	0.205

**Table 3:** Comparison between the time of flight (s) obtained by analyzing the sensors’ data and by the video frame data. The resulting jump height is in meters.

Male	MTw	Video	Height
I Test	0.39 s	0.42 s	0.29
II Test	0.39 s	0.42 s	0.22
III Test	0.43 s	0.44 s	0.25
**Female**	**BTS**	**Video**	**Height**
I Test	0.42 s	0.42 s	0.37
II Test	0.40 s	0.42 s	0.40
III Test	0.44 s	0.44 s	0.44
**Male**	**BTS**	**Video**	**Height**
I Test	0.37 s	0.37 s	0.31
II Test	0.39 s	0.39 s	0.15
III Test	0.41 s	0.44 s	0.31
**Female**	**BTS**	**Video**	**Height**
I Test	0.42 s	0.44 s	0.27
II Test	0.41 s	0.42 s	0.23
III Test	0.42 s	0.42 s	0.39
**Male**	**WIVA**	**Video**	**Height**
I Test	0.43 s	0.41 s	0.44
II Test	0.42 s	0.40 s	0.45
III Test	0.41 s	0.40 s	0.35

**Table 4:** Data obtained from the muscle stiffness test, using the MTw and BTS sensors.

Male Stiffness	MTw	BTS
*k* Minimum	44.56 kN	33.58 kN
*k* Mean	44.75 kN	40.88 kN
*k* Maximum	55.75 kN	54.83 kN
*w* Minimum	1.38 s${}^{-1}$	1.5 s^−1^
*w* Mean	1.66 s${}^{-1}$	1.72 s^−1^
*w* Maximum	2.01 s${}^{-1}$	2.01 s^−1^
*k* Minimum	22.84 kN	25.61 kN
*k* Mean	28.83 kN	35.69 kN
*k* Maximum	37.76 kN	74.02 kN
*w* Minimum	1.75 s${}^{-1}$	1.25 s^−1^
*w* Mean	2.04 s${}^{-1}$	1.88 s^−1^
*w* Maximum	2.26 s${}^{-1}$	2.13 s^−1^

## Abbreviations

The following abbreviations are used in this manuscript:

IMU	Inertial measurement unit
IMMU	Inertial-magnetic measurement unit
SDI	Strap-down integration
CMJ	Counter-movement jump
SL	Step length
IPM	Inverted pendulum model
SITS	Sit-to-stand time
SSTS	Stand-to-sit time
TA	Turn-around time
TOF	Time of flight
TUG	Timed Up and Go
